# Research trends of transcutaneous electrical nerve stimulation in pain management from 2015 to 2024: A bibliometric and visualization analysis

**DOI:** 10.1097/MD.0000000000049900

**Published:** 2026-07-24

**Authors:** Xukang Feng, Yanling Zhou, Yao Yang, Jiafeng Peng, Hongxing Zhang, Junchen Zhu

**Affiliations:** a Department of Orthopaedics, The Second Affiliated Hospital of Anhui University of Chinese Medicine, Hefei, Anhui Province, China; b Department of Second Clinical Medical College, Anhui University of Chinese Medicine, Hefei, Anhui Province, China.

**Keywords:** bibliometric analysis, CiteSpace, pain, TENS, visualization, VOSviewer

## Abstract

**Background:**

Transcutaneous electrical nerve stimulation (TENS) is a widely used nonpharmacological neuromodulation therapy for pain management. Although research in this field has expanded substantially, its global research landscape, knowledge structure, and emerging trends have not been systematically evaluated. This study aimed to comprehensively characterize the development of TENS research for pain management using bibliometric and visualization analyses.

**Methods:**

Publications on TENS for pain management published between 2015 and 2024 were retrieved from the Web of Science Core Collection. Bibliometric analyses of countries, institutions, authors, journals, cited references, and keywords were performed using VOSviewer (version 1.6.18) and CiteSpace (version 6.4.R1).

**Results:**

A total of 968 publications were included. Annual scientific output showed an overall upward trend, reaching its highest level in 2024. The United States was the leading contributor in publication output and total citations, whereas England demonstrated the greatest citation impact. The University of Iowa was the most productive institution, whereas Sluka, Kathleen A. was the most influential author. Keyword co-occurrence, reference clustering, and burst analyses indicated that research has evolved from evaluating analgesic efficacy toward disease-specific applications, methodological optimization, mechanistic exploration, multidimensional outcome assessment, and combination therapies. Emerging topics included phantom limb pain, labor pain, preventive applications, and evidence synthesis.

**Conclusion:**

Research on TENS for pain management has grown steadily over the past decade, with increasing emphasis on methodological rigor and diversified clinical applications. This study provides a comprehensive overview of the field and offers valuable insights to guide future research and evidence-based clinical practice.

## 1. Introduction

Pain is a highly prevalent condition globally, defined by the International Association for the Study of Pain as “an unpleasant sensory and emotional experience associated with, or resembling that associated with, actual or potential tissue damage.” Epidemiological studies indicate that approximately 20% of the world’s population is affected by pain annually.^[1,2]^ Notably, pain substantially impairs patients’ quality of life and is associated with healthcare expenditures that exceed those of heart disease and cancer, thereby imposing a significant economic burden on both families and society.^[3,4]^ Currently, pharmacological interventions provide rapid pain alleviation in the short term, yet extended administration may lead to significant adverse effects.^[5–7]^ These limitations underscore the demand for accessible nonpharmacological alternatives suitable for first-line management, particularly modalities characterized by safety, cost-effectiveness, and ease of administration.

As a neuromodulation therapy, transcutaneous electrical nerve stimulation (TENS) is also commonly used to treat different categories of pain.^[8,9]^ Owing to its low cost, favorable safety profile, and ease of use, TENS is regarded as particularly suitable for integration into community-based and primary care settings.^[10]^ To the best of our knowledge, the analgesic mechanisms of TENS are multidimensional. Previous evidence identified that the large-diameter afferent fibers (Aβ) can inhibit nociceptive transmission according to the gating theory.^[11]^ TENS with high-frequency and low-intensity produces stimulation that selectively activates large-diameter afferent fibers to induce analgesic effects, whereas TENS with low-frequency and high-intensity can activate the descending pain inhibitory pathway by enhancing small-diameter afferent fiber activity.^[12]^ In addition, different frequencies of TENS can exert analgesic effects by activating specific endogenous opioid receptors.^[13]^

Bibliometric analysis has been increasingly utilized as a quantitative research method in the field of pain studies in recent years, serving to delineate disciplinary trends and inform scientific planning.^[14,15]^ By examining bibliographic data – including publication outputs, countries, institutions, authors, journals, and keywords – with the aid of specialized tools such as VOSviewer and CiteSpace, researchers can visualize intellectual structures and track the evolution of research fronts.^[16]^ Furthermore, techniques such as co-citation and co-occurrence analysis facilitate the identification of influential references and emerging thematic clusters, thereby offering a macroscopic perspective on the knowledge architecture of a scientific domain.^[17]^

Although the volume of publications on TENS for pain management has increased substantially in recent years, a comprehensive bibliometric synthesis of this literature has not yet been conducted. To address this gap, a systematic bibliometric analysis was performed to examine the scientific output related to TENS and pain over the past decade. This study aimed to map the current research landscape, identify leading contributors, and detect emerging trends and research hotspots. The findings are anticipated to offer valuable insights for researchers, clinicians, and policymakers, while also helping to direct future scientific efforts in this field.

## 2. Materials and methods

### 2.1. Data collection

The publications were retrieved from the Web of Science Core Collection (WoSCC) on June 20, 2025, and the search strategy is shown in Table 1. WoSCC was selected as the sole data source because it is one of the most widely used and authoritative multidisciplinary citation databases for bibliometric research. It provides standardized bibliographic records, complete citation information, and reliable metadata, which are essential for co-authorship, co-citation, keyword co-occurrence, and citation burst analyses using VOSviewer and CiteSpace. In addition, using a single database can help reduce inconsistencies caused by differences in indexing rules, citation formats, and duplicate records across multiple databases.

**Table 1:** Details of the search strategy for WoSCC.

Number	Search terms
1	TS = (“transcutaneous electrical nerve stimulation”)
2	TS = (pain* OR ache* OR analg* OR nocicep* OR headache* OR head-ache* OR migraine* OR “stomach ache” OR “tummy ache*” OR “abdominal ache*” OR “belly ache*” OR earache* OR “ear ache*” OR toothache* OR tooth-ache* OR odontalgi* OR dysmenorrh* OR neuralgi* OR radiculalg* OR “colic or sciatic*” OR arthritis OR osteoarthritis OR fibromyalg* OR hyperalg* OR ophthalmodyn* OR cephalalg*)
3	1 AND 2

TS = title, abstract, author keywords, and keywords plus, WoSCC = Web of Science Core Collection, * = any ending to the word.

**Figure 1.**
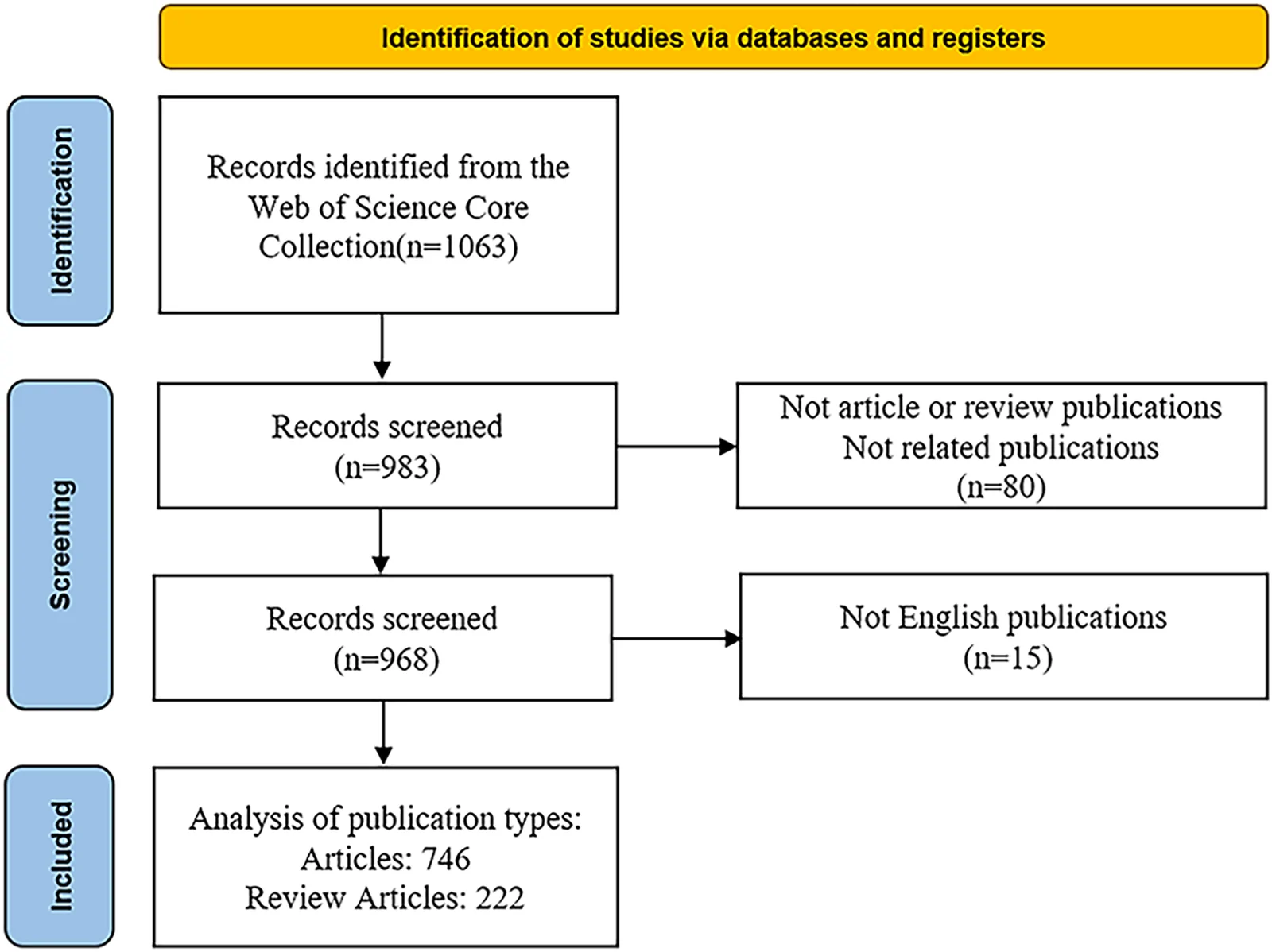
The searching and screening process in WoSCC. WoSCC = Web of Science Core Collection.

All publications met the following inclusion criteria: the timespan was from 2015 to 2024; publication types included articles and reviews; and the publication language was English. All retrieved records were imported into CiteSpace for duplicate checking before bibliometric analysis, and no duplicate records were identified. The flowchart for searching and screening is shown in Figure 1.

### 2.2. Data analysis

The searching and screening of all publications retrieved from WoSCC were carried out independently by 2 authors, and any disagreements were adjudicated by a third author. Bibliometric information was then extracted from the eligible records, including countries, institutions, authors, journals, citation counts, cited references, keywords, Impact Factor (IF), and Hirsch index (H-index). The H-index was used as an indicator to evaluate the academic influence of authors within the retrieved dataset.^[18]^ Before bibliometric analysis, data standardization was performed to improve the accuracy and reliability of the results. Keyword normalization was conducted by merging synonymous terms, abbreviations, spelling variants, and singular/plural forms. For example, terms referring to TENS were standardized where appropriate. In addition, country, institution, and author names were manually checked to reduce ambiguity caused by different spelling formats, abbreviations, or institutional name variants. Any uncertainties or discrepancies were discussed by 2 authors and adjudicated by a third author when necessary. After duplicate checking and data standardization, the final dataset was used for bibliometric and visualization analyses in VOSviewer and CiteSpace.

VOSviewer version 1.6.18 was used to analyze institutional and author collaboration networks.^[19]^ Nodes represent distinct analytical entities, such as institutions or authors, with larger nodes indicating higher publication output. The links between nodes represent collaborative relationships, and thicker links indicate stronger collaboration strength. The counting method was set as full counting. For the institutional collaboration network, the minimum number of documents of an institution was set to 5. For the author collaboration network, the minimum number of documents of an author was set to 3. Based on the collaborative network data generated by VOSviewer, Charticulator (https://donghaoren.org/charticulator/index.html) was further used to construct a chord diagram for visualizing institutional collaborations.

CiteSpace version 6.4.R1 was used to analyze country collaboration networks, cited-reference co-citation networks, keyword co-occurrence networks, reference clusters, and keyword bursts.^[20]^ In the generated maps, node size represents frequency or citation counts, node color indicates publication year, and links denote collaboration, co-occurrence, or co-citation relationships. Nodes with purple outer rings indicate high betweenness centrality and are generally regarded as pivotal points in the network. The time span was set from January 2015 to December 2024, with 1 year per slice. The link strength was calculated using the cosine method, and the scope was set as within slices. For the country collaboration, cited-reference co-citation, and keyword co-occurrence analyses, the node types were set as Country, Reference, and Keyword, respectively. For all 3 analyses, the *g*-index was used as the selection criterion with a scale factor of *k* = 15, and Pathfinder and pruning sliced networks were applied. For both reference and keyword clustering analyses, cluster labels were extracted using the log-likelihood ratio algorithm. Keyword burst detection was performed using the burst detection function of CiteSpace to identify terms with rapidly increasing research attention during the study period.

### 2.3. Ethics section

Ethics approval and informed consent were not required because this bibliometric study analyzed publicly available bibliographic records and did not involve human participants, animals, biological samples, or identifiable personal data.

## 3. Results

### 3.1. Analysis of the annual number of publications

**Figure 2.**
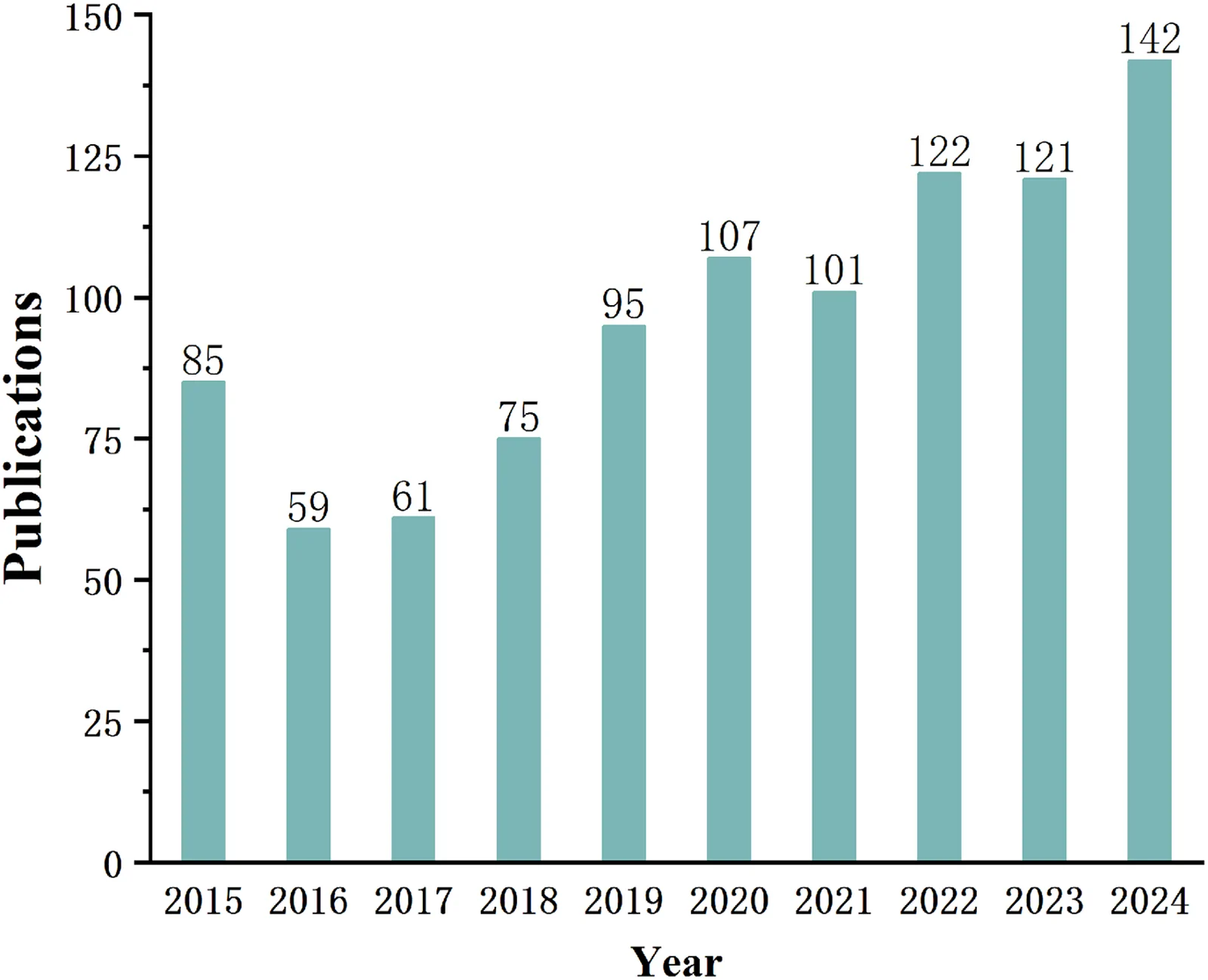
The annual number of publications in this field from 2015 to 2024.

A total of 968 publications from the WoSCC were included in this bibliometric analysis, including 746 articles and 222 reviews. As shown in Figure 2, the annual publication output related to TENS for pain management showed an overall upward trend from 2015 to 2024, despite some fluctuations. Specifically, the number of publications decreased from 2015 to 2016, increased steadily from 2016 to 2020, declined modestly in 2021, and then generally increased thereafter, reaching its highest level in 2024. This trend indicates growing research interest in the application of TENS for pain management.

### 3.2. Analysis of countries

The analysis included 968 publications from 76 countries engaged in TENS research for pain management. The United States made the largest contribution, with 195 publications, followed by China (129 publications), Turkiye (113 publications), Brazil (85 publications), and England (69 publications). As shown in Table 2, the United States accounted for 20.14% of the total publications and accumulated the highest number of citations, with 4366 citations. England and Canada showed relatively high research impact, as reflected by their higher citation-per-paper ratios.

**Table 2:** Top 10 countries with the most publications on TENS for pain management from 2015 to 2024.

Countries	Counts	Percentage	Citations	Citations per paper	Centrality
USA	195	20.14%	4366	22.39	0.34
China	129	13.33%	1912	14.82	0.14
Turkiye	113	11.67%	982	8.69	0.01
Brazil	85	8.78%	1094	12.87	0.09
England	69	7.13%	1813	26.28	0.55
India	64	6.61%	383	5.98	0.10
Canada	52	5.37%	1332	25.62	0.06
Iran	51	5.27%	362	7.10	0.01
Spain	37	3.82%	420	11.35	0.03
South Korea	33	3.41%	539	16.33	0.00

TENS = transcutaneous electrical nerve stimulation.

**Figure 3.**
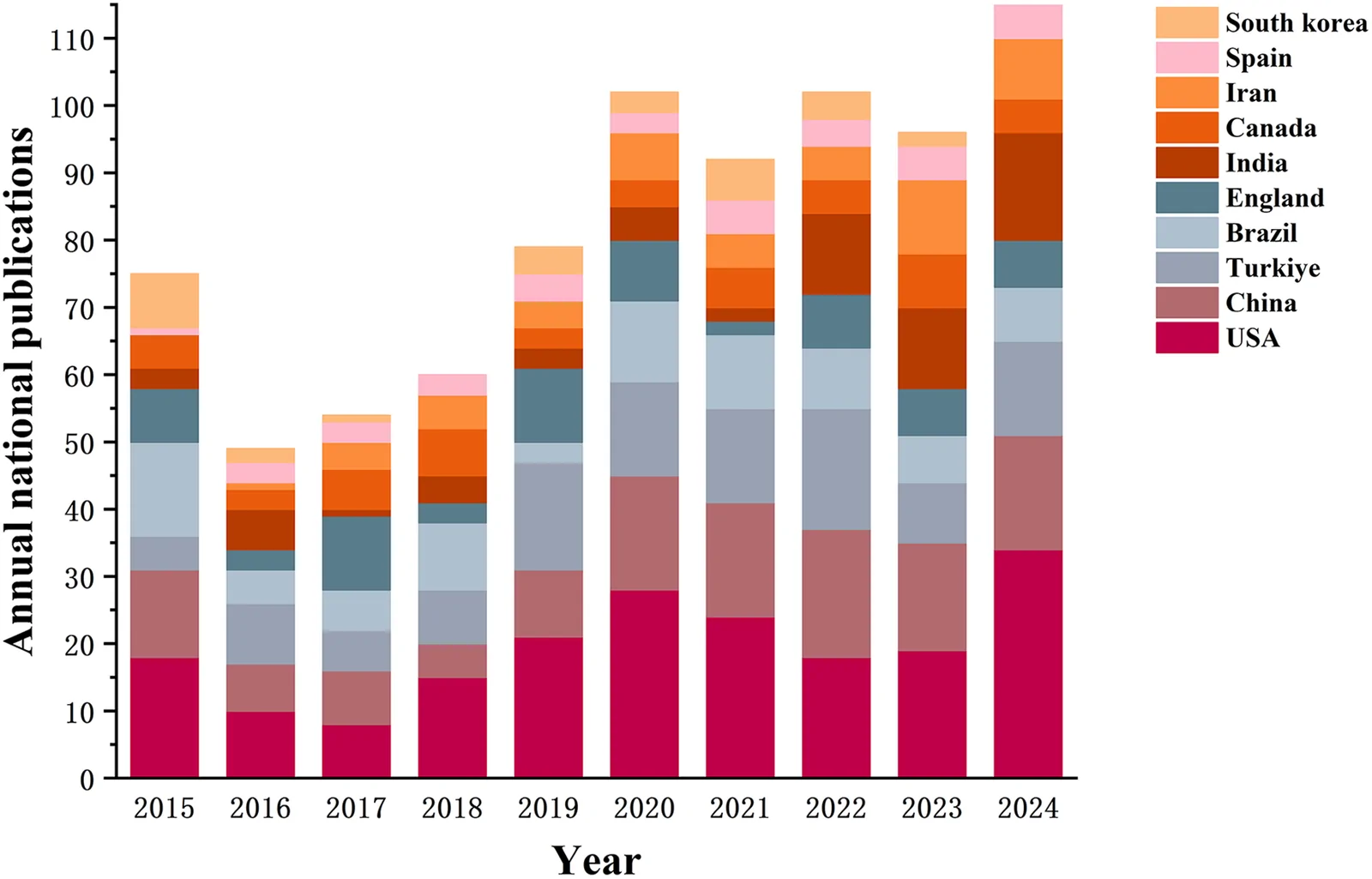
The annual national publication outputs of the top 10 countries.

As illustrated in Figure 3, the United States maintained an overall leading position in annual publication output during the study period, reaching a peak of 34 publications in 2024. Several countries, particularly China, India, and Iran, showed an overall increase in annual publication volume despite fluctuations, indicating their increasing research activity in this field.

**Figure 4.**
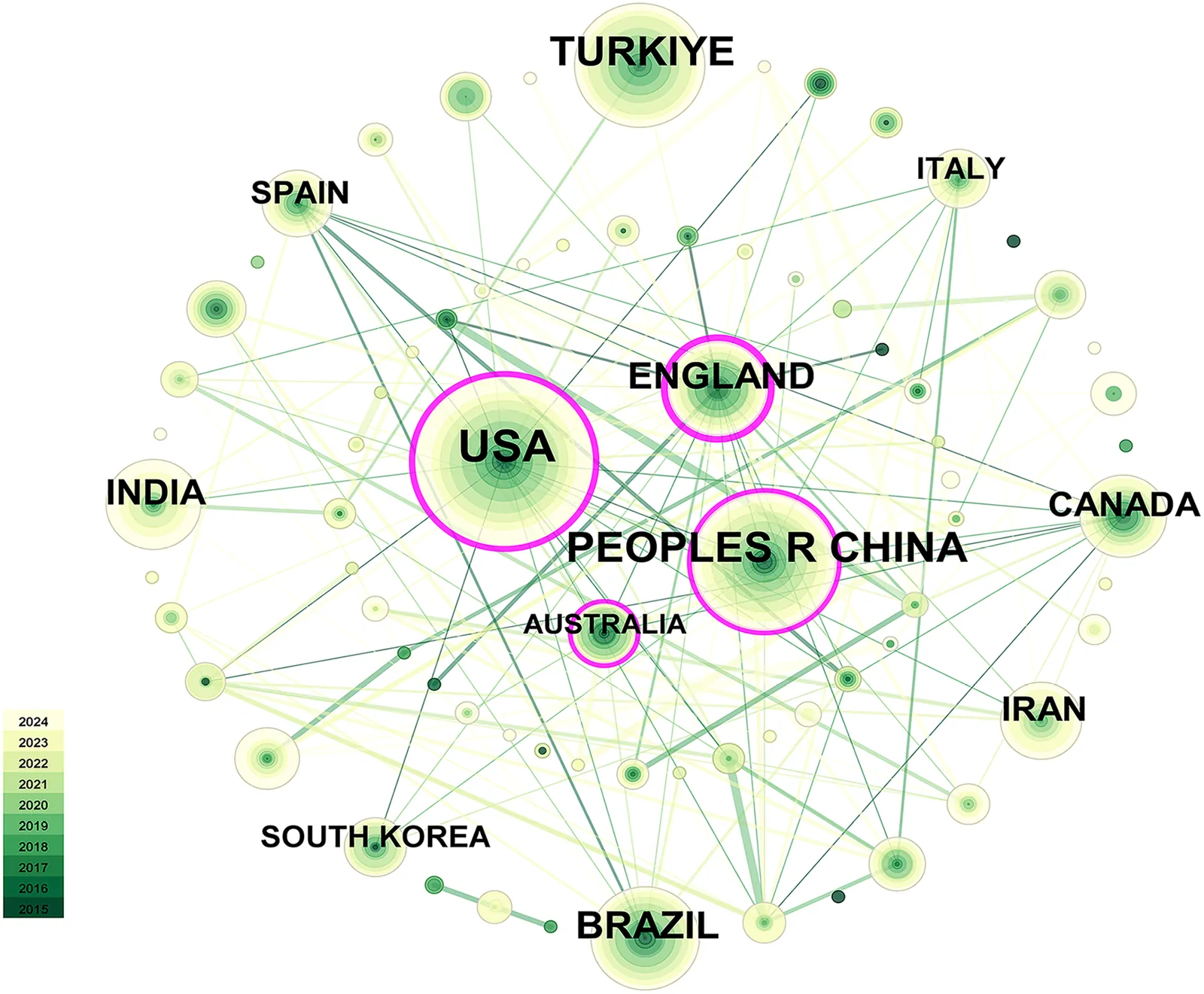
Visualization of country cooperation links in this field from 2015 to 2024.

Figure 4 presents the country collaboration network. The United States (0.34) and England (0.55) had high centrality scores, suggesting that they served as important hubs in international collaboration and knowledge exchange. Although Brazil and Turkiye produced substantial numbers of publications, their lower centrality values indicate that their international collaborative networks remain relatively limited.

### 3.3. Analysis of institutions

The 968 publications involved a total of 1686 institutions. The top 10 institutions by publication output are summarized in Table 3. The University of Iowa ranked first with 24 publications, followed by Cairo University (19 publications), the Federal University of São Carlos (19 publications), and the University of São Paulo (19 publications). Leeds Beckett University showed the highest citation-per-paper ratio (37.75) among the top institutions, indicating a relatively high average citation impact of its research output.

**Table 3:** Top 10 institutions with the most publications on TENS for pain management from 2015 to 2024.

Institutions	Counts	Percentage	Citations	Citations per paper
University of Iowa	24	2.48%	778	32.42
Cairo University	19	1.96%	121	6.37
Federal University of São Carlos	19	1.96%	289	15.21
University of São Paulo	19	1.96%	374	19.68
China Medical University	15	1.55%	152	10.13
Tehran University of Medical Sciences	14	1.45%	93	6.64
Başkent University	12	1.24%	66	5.50
Leeds Beckett University	12	1.24%	453	37.75
University of Toronto	12	1.24%	396	33.00
Shahid Beheshti University of Medical Sciences	11	1.14%	98	8.91

TENS = transcutaneous electrical nerve stimulation.

**Figure 5.**
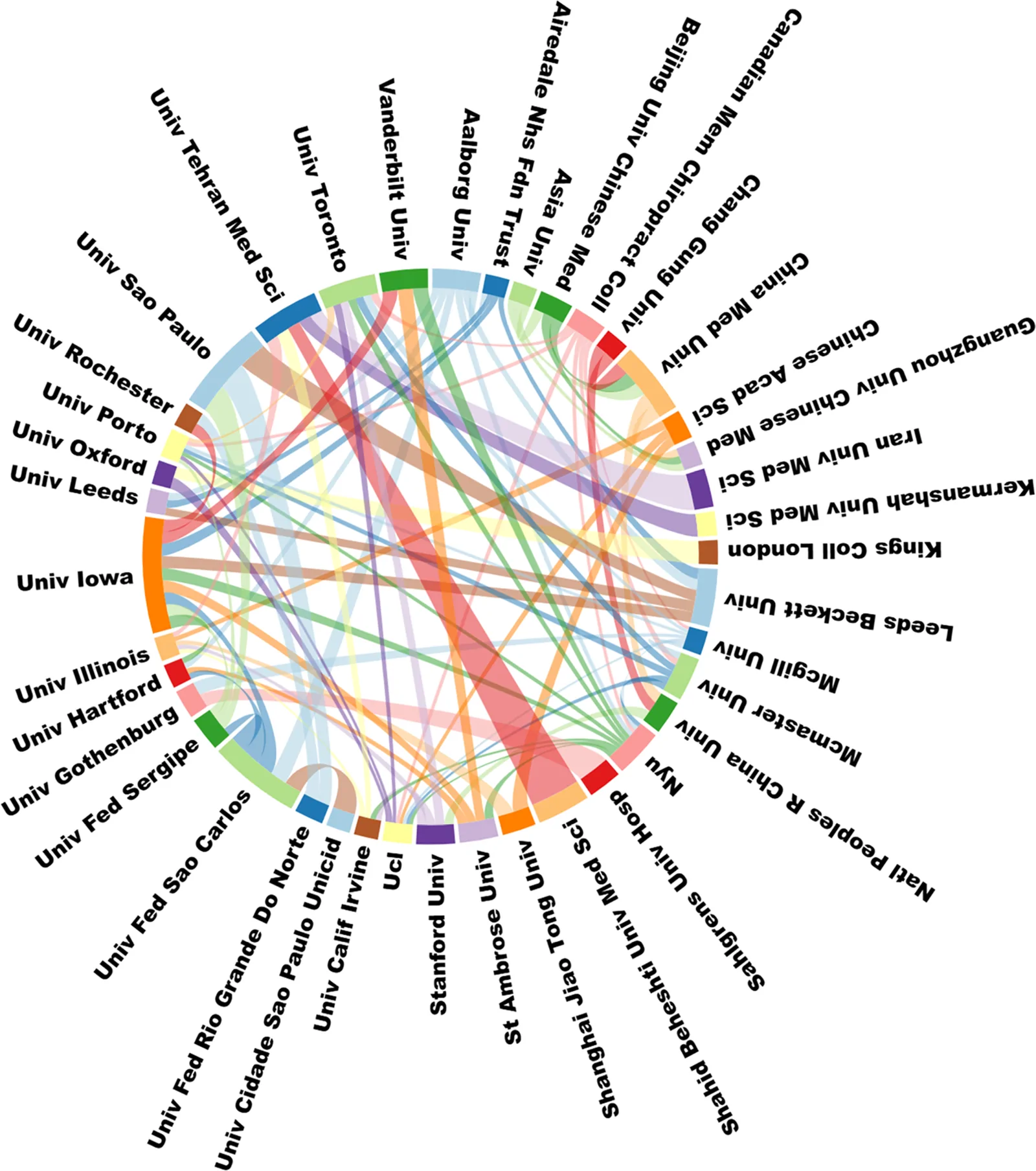
Visualization of institutional cooperation links in this field from 2015 to 2024.

As illustrated in Figure 5, the collaboration network analysis was conducted among institutions with at least 5 publications. Overall, interinstitutional collaboration remained relatively limited. The University of Iowa not only ranked first in publication output but also showed relatively extensive collaboration links in the institutional collaboration network.

### 3.4. Analysis of authors

A total of 4507 authors published articles related to TENS for pain management. The top 10 authors by publication output are shown in Table 4. Liebano, Richard E. was the most prolific author, with 21 publications, followed by Sluka, Kathleen A. (20 publications) and Vance, Carol G. T. (13 publications). Sluka, Kathleen A. had the highest number of citations and the highest H-index, while Johnson, Mark I. showed the highest citation-per-paper ratio (39.00), indicating a relatively high average citation impact.

**Table 4:** Top 10 authors with the most publications on TENS for pain management from 2015 to 2024.

Authors	Counts	Citations	Citations per paper	H-index
Liebano, Richard E	21	217	10.33	8
Sluka, Kathleen A	20	675	33.75	12
Vance, Carol G. T	13	287	22.08	10
Dailey, Dana L	13	276	21.23	9
Chimenti, Ruth L	11	414	37.64	6
Crofford, Leslie J	11	263	23.91	8
Johnson, Mark I	10	390	39.00	8
Rakel, Barbara A	10	224	22.40	8
Leonard, Guillaume	9	80	8.89	5
Zimmerman, M. Bridget	8	212	26.50	7

H-index = Hirsch index, TENS = transcutaneous electrical nerve stimulation.

**Figure 6.**
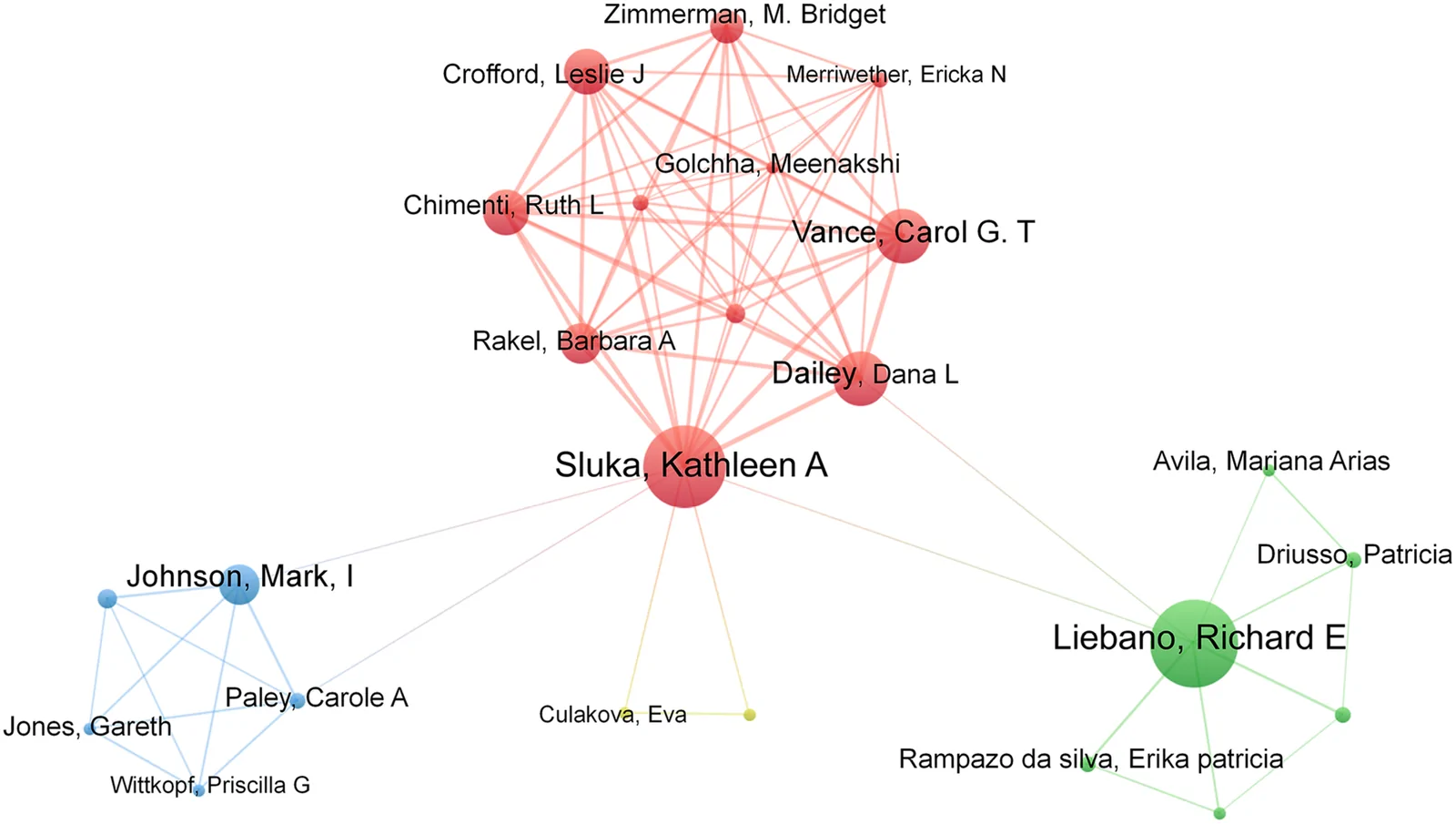
Visualization of author cooperation links in this field from 2015 to 2024.

Figure 6 presents the collaboration network of core authors, defined as authors with 3 or more publications. Several collaborative groups were identified, but relatively few links were observed between different groups, suggesting that author collaboration was mainly concentrated within individual research teams.

### 3.5. Analysis of journals

A total of 427 journals published articles related to TENS for pain management. The top 10 journals by publication output are shown in Table 5. *Neuromodulation* was the most prolific journal, with 22 publications, followed by the *Cochrane Database of Systematic Reviews* (19 publications) and the *Journal of Back and Musculoskeletal Rehabilitation* (18 publications). Among the top journals, the *Cochrane Database of Systematic Reviews* had the highest citation-per-paper ratio (47.32) and the highest 5-year IF (IF = 10.3).

**Table 5:** Top 10 journals with the most publications on TENS for pain management from 2015 to 2024.

Journals	Counts	Citations	Citations per paper	IF (5 years)
*Neuromodulation*	22	241	10.95	3.5
*Cochrane Database of Systematic Reviews*	19	899	47.32	10.3
*Journal of Back and Musculoskeletal Rehabilitation*	18	196	10.89	1.7
*Cureus Journal of Medical Science*	17	35	2.06	1.3
*Medicine*	15	104	6.93	1.6
*Physical Therapy*	15	299	19.93	3.9
*Journal of Pain Research*	14	149	10.64	2.8
*Pain Research Management*	11	183	16.64	3.1
*American Journal of Physical Medicine Rehabilitation*	10	98	9.80	2.4
*European Journal of Pain*	10	132	13.20	3.9

IF = impact factor, TENS = transcutaneous electrical nerve stimulation.

### 3.6. Analysis of cited references

**Figure 7.**
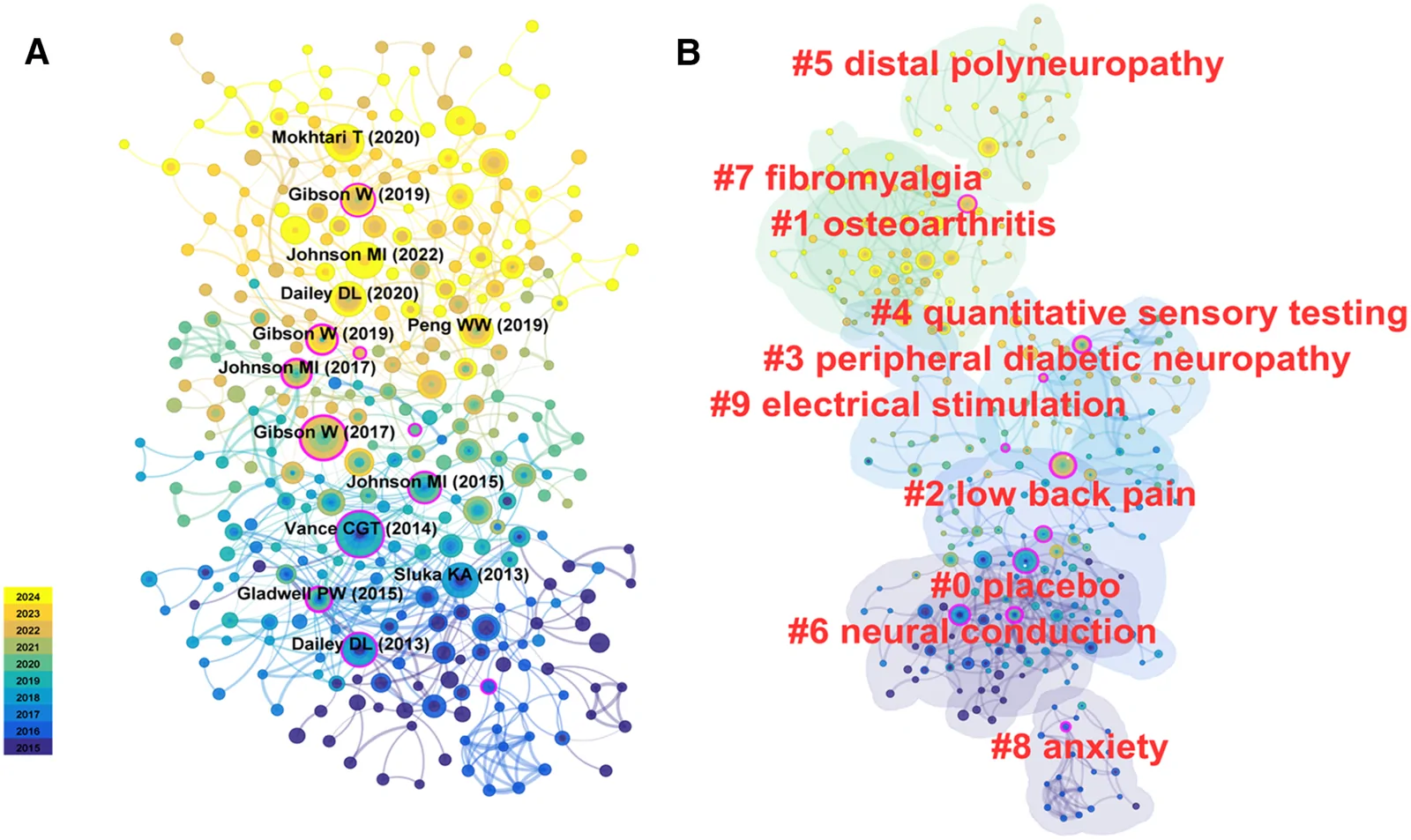
(A) Visualization of cited references related to TENS for pain management from 2015 to 2024. (B) The top 10 clusters of cited references related to TENS for pain management from 2015 to 2024. TENS = transcutaneous electrical nerve stimulation.

Figure 7A shows the cited-reference co-citation network, and Table 6 summarizes the 5 most frequently cited references. The most cited reference was Gibson et al^[21]^, which systematically evaluated 15 randomized controlled trials (RCTs) investigating TENS for neuropathic pain management. The most recent among the highly cited references was the meta-TENS study, which included 381 RCTs and provided more comprehensive evidence that TENS significantly reduced pain intensity both during and after treatment.^[22]^ In addition, several highly cited references focused on the analgesic mechanisms, efficacy determinants, clinical applications, and trial design considerations of TENS.^[23–25]^ As shown in Table 7, the study by Gladwell et al^[26]^, published in *Physical Therapy*, exhibited the highest centrality, suggesting its important bridging role in the co-citation network.

**Table 6:** The top 5 most frequently cited references on TENS for pain management from 2015 to 2024.

Reference	Representative author (publication year)	Frequency
Transcutaneous electrical nerve stimulation (TENS) for neuropathic pain in adults	Gibson W (2017)	38
Using TENS for pain control: the state of the evidence	Vance CGT (2014)	36
Transcutaneous electrical nerve stimulation in relieving neuropathic pain: basic mechanisms and clinical applications	Mokhtari T (2020)	29
What makes transcutaneous electrical nerve stimulation work? Making sense of the mixed results in the clinical literature	Sluka KA (2013)	27
Efficacy and safety of transcutaneous electrical nerve stimulation (TENS) for acute and chronic pain in adults: a systematic review and meta-analysis of 381 studies (the meta-TENS study)	Johnson MI (2022)	27

TENS = transcutaneous electrical nerve stimulation.

**Table 7:** Top 5 centralities of the cited references on TENS for pain management from 2015 to 2024.

Reference	Representative author (publication year)	Centrality
Direct and indirect benefits reported by users of transcutaneous electrical nerve stimulation for chronic musculoskeletal pain: qualitative exploration using patient interviews	Gladwell PW (2015)	0.38
Transcutaneous electrical nerve stimulation (TENS) for fibromyalgia in adults	Johnson MI (2017)	0.28
Transcutaneous electrical nerve stimulation (TENS) for neuropathic pain in adults	Gibson W (2017)	0.23
Transcutaneous electrical nerve stimulation (TENS) for chronic pain – an overview of Cochrane Reviews	Gibson W (2019)	0.20
Using TENS for pain control: the state of the evidence	Vance CGT (2014)	0.19

TENS = transcutaneous electrical nerve stimulation.

Cited-reference cluster analysis identified 10 clusters, which are visualized in Figure 7B. Table 8 presents the structural characteristics of these clusters, including node counts, silhouette values, mean years, and cluster labels. Clusters with silhouette values above 0.5 are generally considered acceptable, whereas values above 0.7 indicate high reliability.^[27]^ In this study, all major clusters had silhouette values >0.7, indicating reliable clustering results. Among them, cluster #5 (“distal polyneuropathy”) had the highest silhouette value (0.949) and contained 25 nodes. The largest cluster was “placebo,” which contained 49 nodes and had an average publication year of 2013. The temporal distribution of the clusters ranged from 2012 to 2020, with the most recent clusters mainly related to “fibromyalgia” (2020), “distal polyneuropathy” (2020), and “osteoarthritis” (2020).

**Table 8:** The structural characteristics of the cited-references clusters.

Cluster	Size	Silhouette	Mean year	Cluster label
#0	49	0.853	2013	Placebo
#1	44	0.871	2020	Osteoarthritis
#2	35	0.930	2015	Low back pain
#3	27	0.841	2018	Peripheral diabetic neuropathy
#4	25	0.900	2016	Quantitative sensory testing
#5	25	0.949	2020	Distal polyneuropathy
#6	25	0.804	2013	Neural conduction
#7	21	0.923	2020	Fibromyalgia
#8	20	0.936	2012	Anxiety
#9	15	0.889	2016	Electrical stimulation

### 3.7. Analysis of keywords

Table 9 presents the top 10 most frequently occurring keywords related to TENS in pain management research. “Transcutaneous electrical nerve stimulation” was the most frequent keyword (549 occurrences), followed by “management” (190 occurrences), “pain” (149 occurrences), “low back pain” (117 occurrences), and “efficacy” (95 occurrences).

**Table 9:** Top 10 keywords in terms of frequency on TENS for pain management from 2015 to 2024.

Rank	Keyword	Frequency
1	Transcutaneous electrical nerve stimulation	549
2	Management	190
3	Pain	149
4	Low back pain	117
5	Efficacy	95
6	Double blind	86
7	Neuropathic pain	76
8	Chronic pain	70
9	Mechanisms	68
10	Quality of life	68

TENS = transcutaneous electrical nerve stimulation.

**Figure 8.**
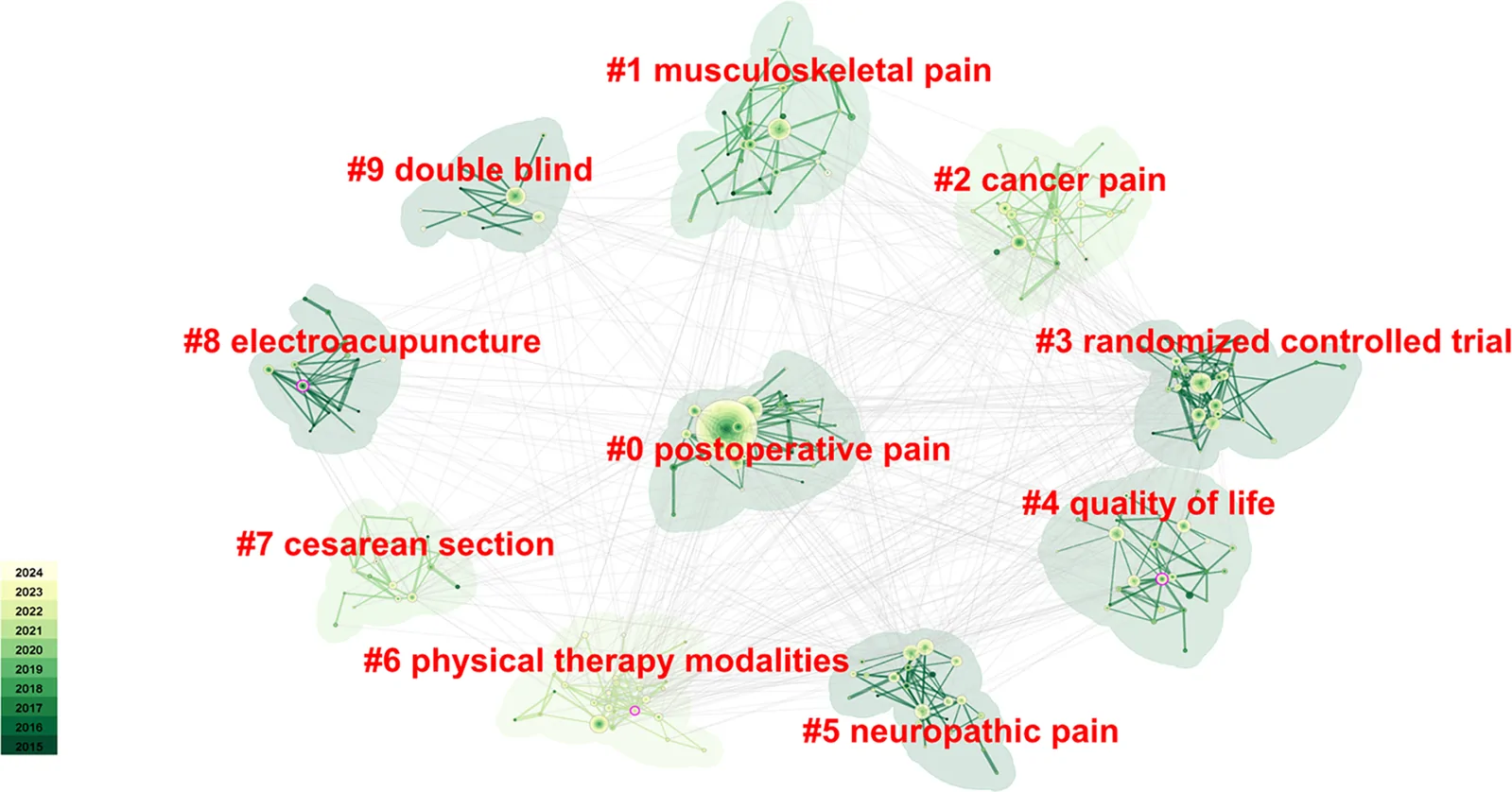
The top 10 clusters of keywords related to TENS for pain management from 2015 to 2024. TENS = transcutaneous electrical nerve stimulation.

Figure 8 shows the keyword co-occurrence clustering results for TENS applications in pain management. Cluster #0, labeled “postoperative pain,” was the largest cluster, containing 36 keywords, followed by “musculoskeletal pain” (33 keywords), “cancer pain” (32 keywords), “randomized controlled trial” (28 keywords), and “quality of life” (26 keywords). Cluster #7, labeled “cesarean section,” was also identified as a relatively recent topic in the keyword clustering map.

**Figure 9.**
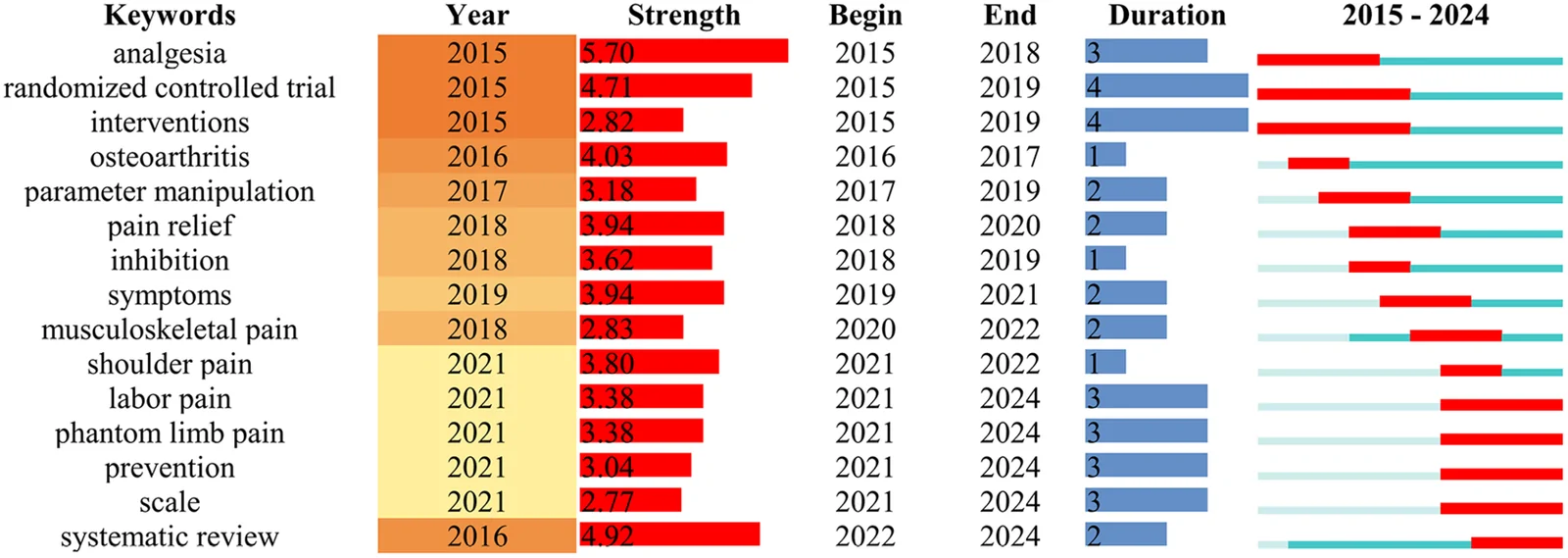
Top 15 keywords with the strongest citation bursts.

Keyword burst detection identified terms with rapidly increasing research attention from 2015 to 2024 (Fig. 9). “Analgesia” showed the strongest burst intensity (strength: 5.70), lasting from 2015 to 2018. Methodological terms, including “randomized controlled trial” (2015–2019) and “systematic review” (2022–2024), also showed notable bursts, reflecting continued attention to evidence quality. Recent burst keywords, including “phantom limb pain” (2021–2024), “labor pain” (2021–2024), “prevention” (2021–2024), and “scale” (2021–2024), may represent emerging research directions in this field.

## 4. Discussion

Previous reviews and meta-analyses have evaluated the clinical efficacy and safety of TENS for different pain conditions.^[28–31]^ However, a bibliometric analysis that systematically maps the overall research landscape, collaboration patterns, knowledge structure, and emerging trends in this field remains lacking. To address this gap, the present study analyzed publications related to TENS for pain management from 2015 to 2024 using bibliometric and visualization methods. The results revealed a growing research interest in this field and identified the major contributing countries, institutions, authors, journals, highly cited references, research hotspots, and emerging frontiers.

### 4.1. General information

The temporal distribution of publications indicates a sustained increase in research attention to TENS for pain management over the past decade. Annual publication output exceeded 100 articles in 2020 and reached its highest level in 2024, suggesting that TENS has remained an active topic in pain research. This growth may reflect increasing interest in nonpharmacological pain management strategies and the expanding clinical application of neuromodulation-based interventions. However, the fluctuations observed across years also suggest that the development of this field has not been entirely linear, and continued high-quality evidence is still needed to support broader clinical implementation.

The country-level analysis revealed both concentration and diversification in global research output. The United States contributed the largest number of publications and total citations, indicating its leading role in this field. England and Canada showed relatively high citation-per-paper ratios, suggesting that research impact is not determined solely by publication volume but is also related to study quality, methodological rigor, and international visibility. In contrast, countries such as China, Turkiye, India, and Iran showed increasing publication activity, reflecting the broader global expansion of TENS-related research. Nevertheless, some countries with substantial publication output had relatively lower citation impact or centrality, indicating that stronger international collaboration and higher-quality clinical research may be needed to enhance their academic influence.

Institutional and author collaboration patterns further indicate that the field remains relatively fragmented. Although the University of Iowa was the most productive institution and showed active collaboration links, the overall institutional collaboration network was limited. Similarly, several core author groups were identified, but connections between different research teams remained sparse. This pattern suggests that current TENS research is still largely driven by relatively independent groups rather than by broadly connected international networks. Strengthening cross-institutional and cross-national collaboration may help promote standardized stimulation protocols, harmonized outcome measures, and multicenter clinical trials, thereby improving the generalizability and robustness of future evidence.

The author-level analysis also showed a distinction between productivity and academic influence. Liebano, Richard E. was the most prolific author, whereas Sluka, Kathleen A. showed the greatest overall influence based on citation count and H-index. Johnson, Mark I. had the highest citation-per-paper ratio, indicating a high average impact of his publications. These findings suggest that influential contributions in this field are not limited to publication quantity but are also shaped by sustained work on mechanisms, clinical efficacy, evidence synthesis, and methodological development.

The journal distribution reflects the interdisciplinary nature of TENS research. Publications were concentrated in journals related to neuromodulation, rehabilitation, pain medicine, and evidence-based medicine. Neuromodulation published the largest number of articles, while the Cochrane Database of Systematic Reviews showed the highest citation-per-paper ratio and 5-year IF among the top journals. This pattern indicates that both original clinical studies and high-quality evidence syntheses have played important roles in shaping the knowledge base of TENS for pain management. Future studies should not only expand clinical applications but also address evidence gaps identified by systematic reviews through rigorous trial design and standardized reporting.

### 4.2. Research hotspots and frontiers

The integrated analysis of cited-reference clusters, keyword co-occurrence clusters, and burst keywords revealed several major research themes in TENS for pain management. Overall, the field has evolved from broad evaluations of analgesic efficacy toward more specific clinical indications, improved trial methodology, multidimensional outcome assessment, mechanistic exploration, and combined therapeutic strategies.

#### 4.2.1. TENS in different types of pain management

##### 4.2.1.1. Postoperative pain

Postoperative pain is a common clinical problem that may impair quality of life and delay functional recovery.^[32]^ In the present bibliometric analysis, “postoperative pain” was identified as the largest keyword cluster, indicating that perioperative analgesia is one of the major application areas of TENS. Over the past decade, TENS has been investigated in several surgical contexts, including cardiothoracic,^[33]^ abdominal,^[34]^ and orthopedic.^[35]^ The identification of “cesarean section” as a relatively recent keyword cluster further suggests increasing attention to postcesarean pain management, which may be related to the global rise in cesarean section rates.^[36,37]^

Current evidence indicates that TENS may reduce both incisional pain and postpartum uterine contraction pain after cesarean delivery,^[38]^ with some studies reporting more evident analgesic effects within the first 24 hours after surgery.^[39]^ However, findings remain inconsistent, as another randomized trial did not observe significant pain reduction after TENS application.^[40]^ These discrepancies may partly reflect heterogeneity in stimulation parameters, treatment duration, intervention frequency, electrode placement, and the clinical characteristics of surgical populations. Therefore, future studies should place greater emphasis on standardized intervention protocols and clearly reported stimulation parameters.

##### 4.2.1.2. Musculoskeletal pain

Musculoskeletal pain represents a substantial clinical and socioeconomic burden worldwide.^[41]^ In this study, musculoskeletal pain-related topics, including low back pain, osteoarthritis, and fibromyalgia, appeared in both keyword and cited-reference clusters, suggesting that musculoskeletal disorders remain a central focus of TENS research. Emerging evidence from an RCT revealed that TENS significantly alleviated symptoms in patients with preradiographic knee osteoarthritis, suggesting its potential as an early therapeutic intervention.^[42]^ A prospective study further reported a progressive reduction in Visual Analog Scale pain scores among patients with knee osteoarthritis receiving TENS during a 19-month follow-up period.^[43]^

Beyond pain intensity, some studies have explored functional and structural outcomes. For example, lumbar TENS was reported to reduce low back pain and decrease erector spinae muscle thickness, with a correlation between pain reduction and muscle thinning.^[44]^ Fibromyalgia has also received increasing attention in recent years.^[45–47]^ Recent evidence suggests that TENS may reduce movement-evoked pain in patients with fibromyalgia, including those with concurrent opioid use.^[48]^ These findings support the potential role of TENS as a nonpharmacological option for musculoskeletal pain, although its long-term effects and optimal parameters still require further validation.

##### 4.2.1.3. Cancer pain

Cancer pain was also identified as an important keyword cluster, reflecting the application of TENS in supportive and palliative care settings. Some clinical studies have reported analgesic benefits of TENS in cancer-related pain. In patients with pancreatic cancer, TENS was associated with a marked reduction in numeric rating scale scores immediately after treatment, with effects persisting for several weeks.^[49]^ A retrospective cohort study also found clinically meaningful reductions in Visual Analog Scale pain scores among oncology patients receiving TENS therapy, with the study designed to evaluate TENS as a goal-directed intervention for functional improvement.^[50]^ In addition, Nakano et al^[51]^ reported that TENS may improve not only pain but also other symptoms, such as nausea and appetite loss, in patients receiving palliative care. These findings suggest that TENS may have value in multidimensional symptom management for cancer patients. Nevertheless, more rigorous prospective trials are needed to clarify its role across different cancer types, disease stages, and palliative care contexts.

##### 4.2.1.4. Neuropathic pain

Neuropathic pain, defined as pain caused by a lesion or disease of the somatosensory system, is associated with substantial disability and reduced quality of life.^[52]^ The prominence of neuropathic pain-related references and the emergence of the “distal polyneuropathy” cluster indicate sustained interest in TENS for neuropathic conditions. Clinical evidence suggests that TENS may provide benefits in nerve injury-related pain disorders.^[53,54]^ In diabetic peripheral neuropathy, studies have reported improvements in pain intensity after TENS treatment.^[55,56]^ More recent studies have extended this research focus to chemotherapy-induced peripheral neuropathy and alcohol-related peripheral neuropathy.^[57,58]^ These findings suggest that neuropathic pain remains an important and expanding area for TENS research, especially in conditions with limited safe and effective long-term treatment options.

#### 4.2.2. Methodology of clinical trials

The keyword cluster “randomized controlled trial” indicates that methodological rigor has been a major concern in TENS research. Because TENS involves a perceptible sensory stimulus, blinding and placebo control are particularly challenging. A credible sham design is essential to reduce expectation bias among both participants and investigators. Previous studies have used sham devices with no current output or imperceptible electrical stimulation.^[59,60]^ More recently, transient sham TENS devices have been developed to improve blinding. For example, 1 sham device delivers brief stimulation for 30 seconds and then gradually reduces the current intensity to 0 mA over 15 seconds.^[61]^ Such designs may help preserve the credibility of placebo control while minimizing expectancy effects.

The emergence of “randomized controlled trial” and “systematic review” as burst keywords further suggests that the field is moving toward higher standards of evidence generation and synthesis. However, heterogeneity in stimulation frequency, intensity, pulse width, session duration, electrode placement, comparator design, and outcome measurement remains a major challenge. Therefore, future TENS trials should improve the standardization and transparency of intervention reporting, adopt credible sham controls where appropriate, and use clinically meaningful outcome measures.

#### 4.2.3. Holistic therapeutic outcomes

The keyword cluster “quality of life” and the cited-reference cluster “anxiety” suggest that TENS research has expanded beyond pain intensity alone.^[62,63]^ This trend is consistent with the high centrality of the study by Gladwell et al,^[26]^ which reported both direct benefits of TENS, such as pain relief, attentional distraction, and reduced muscle tension, and indirect benefits, including decreased analgesic use, improved function, psychological well-being, and better sleep quality. These findings highlight the importance of evaluating TENS through multidimensional outcome frameworks.

Traditional pain scales are useful but may not fully capture the therapeutic effects of TENS. For patients with chronic pain, outcomes such as physical function, sleep quality, emotional status, medication use, and quality of life may be equally relevant. Therefore, future studies should incorporate patient-centered and multidimensional outcome measures to better evaluate the broader clinical value of TENS.

#### 4.2.4. Analgesic mechanisms

The high frequency of the keyword “mechanisms” indicates sustained interest in the biological basis of TENS analgesia. Current evidence suggests that TENS acts through both peripheral and central mechanisms. At the peripheral level, TENS has been shown to regulate pain-related neuropeptides and neuroinflammatory gene expression in dorsal root ganglia.^[64]^ At the central level, TENS may activate descending inhibitory pathways. Both high- and low-frequency TENS have been reported to modulate opioid receptor-mediated analgesia in the ventrolateral periaqueductal gray and rostral ventromedial medulla.^[65]^ Specifically, high-frequency TENS is associated with δ-opioid receptor activation, whereas low-frequency TENS preferentially activates μ-opioid receptors.^[66]^

Neuroimaging and quantitative sensory testing have further supported the central modulatory effects of TENS. Functional magnetic resonance imaging studies suggest that TENS may alter activity in somatosensory and parietal regions and enhance functional connectivity between the periaqueductal gray and lateral prefrontal cortex.^[67]^ Quantitative sensory testing has shown that TENS can increase pressure pain thresholds and restore conditioned pain modulation, suggesting enhanced central inhibitory function.^[68]^ However, longer-term interventions have not consistently produced parallel changes in conditioned pain modulation, even among clinically responsive patients.^[69]^ These findings suggest that the mechanistic profile of TENS may vary according to treatment duration, stimulation parameters, pain condition, and patient phenotype. Further mechanistic studies are therefore needed to identify biomarkers of response and optimize individualized treatment protocols.

#### 4.2.5. Combined therapeutic modalities

The keyword cluster “physical therapy modalities” reflects increasing interest in combining TENS with other nonpharmacological interventions. For example, TENS combined with physical therapy reduced pain scores and analgesic consumption in male patients with pudendal neuralgia,^[70]^ while TENS combined with stretching improved pain and functional outcomes in myofascial pain syndrome.^[71]^ In addition, burst TENS combined with cryotherapy increased pressure pain thresholds and tolerance more effectively than either intervention alone.^[72]^

However, combination effects may depend on the specific TENS mode and the accompanying therapy. Macedo et al^[72]^ reported that burst TENS, but not conventional TENS, showed synergistic effects when combined with cryotherapy. This finding suggests that interactions between stimulation parameters and adjunctive therapies should be carefully considered. Future studies should move beyond simply combining interventions and instead examine which combinations are mechanistically compatible, clinically effective, and feasible for routine care.

The relationship between TENS, electroacupuncture (EA), and transcutaneous electrical acupoint stimulation (TEAS) also deserves attention. Both TENS and EA exert neuromodulatory effects and have been applied in pain management.^[30,73]^ Compared with EA, TENS is noninvasive and more suitable for self-administration.^[74,75]^ TEAS combines the noninvasive features of TENS with acupoint-based stimulation and has become an emerging modality in analgesia-related research.^[76]^ Neuroimaging studies suggest that EA and TEAS may produce different central regulatory patterns,^[77]^ possibly involving brain regions related to sensory, affective, and cognitive components of pain.^[78]^ However, much of the current mechanistic evidence is derived from healthy participants. Further clinical studies in pain populations are needed to clarify the comparative efficacy, mechanisms, and optimal indications of TENS, EA, and TEAS.

### 4.3. Future perspectives

Burst keyword analysis provides insight into emerging directions in TENS research. The recent bursts of “labor pain” and “phantom limb pain” suggest that the clinical indications of TENS are expanding beyond conventional musculoskeletal and postoperative pain conditions. Future studies may focus on optimizing stimulation parameters, such as frequency, pulse width, and intensity, for phantom limb pain,^[79,80]^ as well as developing individualized protocols for labor pain that consider differences in pain perception and obstetric characteristics.^[81]^

The burst keyword “prevention” indicates growing interest in the prophylactic potential of TENS, particularly in areas such as migraine and chronic postoperative pain.^[82,83]^ This suggests a possible shift from using TENS solely as a symptomatic analgesic intervention toward exploring its preventive applications. However, preventive use requires stronger evidence from well-designed prospective trials with clearly defined populations, intervention timing, and long-term outcomes.

The recent burst of “systematic review” during 2022 to 2024 indicates that the field is increasingly emphasizing evidence synthesis.^[22,84–86]^ This trend is important because the existing TENS literature remains heterogeneous in terms of pain conditions, stimulation protocols, comparator groups, and outcome measures. High-quality systematic reviews can help identify evidence gaps, but they also depend on the methodological quality of the original studies. Finally, the emergence of “scale” as a burst keyword reflects continued concern about sample size, measurement tools, and methodological rigor.^[12,87]^ Future research should therefore prioritize large-scale, multicenter RCTs, standardized reporting of stimulation parameters, credible sham controls, and multidimensional outcome assessment. These efforts will be essential for improving the quality of evidence and guiding the clinical application of TENS in pain management.

## 5. Strengths and limitations

This study has several strengths. It provides a comprehensive and systematic bibliometric overview of the global research landscape, collaborative networks, knowledge structure, and emerging trends in TENS for pain management over the past decade. By using VOSviewer and CiteSpace, we visualized collaboration networks, reference co-citation networks, keyword co-occurrence patterns, and burst keywords, thereby identifying major contributors, knowledge structures, research hotspots, and emerging trends in this field. These findings provide a broad overview of the development of TENS research and may help researchers better understand current priorities and potential future directions.

However, several limitations should be acknowledged. First, although WoSCC is a widely recognized multidisciplinary database with high-quality citation records and standardized bibliographic information, the exclusive use of WoSCC may have introduced database selection bias. Relevant publications indexed only in other databases, such as Scopus, PubMed, Embase, or Google Scholar, may have been omitted. In particular, clinically relevant studies, regional publications, and non-English literature may not have been fully captured. Therefore, the findings should be interpreted as reflecting the research landscape of TENS for pain management based on WoSCC-indexed publications rather than the entire body of global literature. Second, the analysis was restricted to English-language articles and reviews published from 2015 to 2024. As a result, non-English studies and publications from 2025 were not included, which may have led to the omission of very recent developments. Third, bibliometric results may be influenced by citation time lag, database indexing practices, and software parameter settings. Although we standardized the data and reported the main parameters used in VOSviewer and CiteSpace, the results should still be interpreted with these methodological constraints in mind. Despite these limitations, this study provides a timely and structured overview of the research evolution, current hotspots, and emerging frontiers of TENS for pain management.

## 6. Conclusion

This study provides a comprehensive bibliometric and visualization analysis of global research on TENS for pain management from 2015 to 2024, offering an integrated overview of its research landscape, knowledge structure, and emerging trends. The findings demonstrate sustained growth in scientific output and identify the leading countries, institutions, authors, journals, influential references, and evolving knowledge structure that have shaped this field. The research landscape has gradually evolved from broad evaluations of analgesic efficacy toward more diverse clinical applications, improved methodological rigor, mechanistic exploration, comprehensive outcome assessment, and integrative therapeutic strategies. Nevertheless, relatively limited international collaboration and considerable heterogeneity in stimulation protocols, study design, and outcome measures continue to hinder the generation of robust and generalizable evidence. Future research should therefore prioritize multicenter collaborative studies, standardized intervention and reporting protocols, high-quality RCTs, and patient-centered multidimensional outcome assessment to strengthen the evidence base and facilitate the translation of TENS into clinical practice. Overall, this study provides a comprehensive overview of the current research landscape and may serve as a valuable reference for future research planning, evidence synthesis, and the evidence-based application of TENS in pain management.

## Acknowledgments

We extend our sincere gratitude to all the researchers, institutions, and journals whose contributions in this field have facilitated the completion of this work. Their dedicated efforts and valuable insights have been instrumental in the development of this manuscript.

## Author contributions

**Writing – original draft:** Xukang Feng, Yanling Zhou, Yao Yang.

**Writing – review & editing:** Jiafeng Peng, Hongxing Zhang, Junchen Zhu.

## Correction

This article was originally published with the funding statement Grant No. 0549-6; it has now been corrected online to show Grant No. 202427b10020069.
